# Genetic Risk Score of Nine Type 2 Diabetes Risk Variants that Interact with Erythrocyte Phospholipid Alpha-Linolenic Acid for Type 2 Diabetes in Chinese Hans: A Case-Control Study

**DOI:** 10.3390/nu9040376

**Published:** 2017-04-11

**Authors:** Ju-Sheng Zheng, Kelei Li, Tao Huang, Yanqiu Chen, Hua Xie, Danfeng Xu, Jianqin Sun, Duo Li

**Affiliations:** 1 Department of Food Science and Nutrition, Zhejiang University, Hangzhou 310058, China; zhengjusheng@gmail.com (J.-S.Z.); wfujfqcc@sina.com (K.L.); 2 Institute of Nutrition and Health, Qingdao University, Qingdao 266071, China; 3 Saw Swee Hock School of Public Health, National University of Singapore, Singapore 117549, Singapore; taohuang83@gmail.com; 4 Clinical Nutrition Center, Huadong Hospital, Fudan University, Shanghai 200040, China; jxfbtangx@126.com (Y.C.); yujinghuang.zju@gmail.com (H.X.); jxfbzhus@126.com (D.X.); jianqins@gmail.com (J.S.)

**Keywords:** n-3 fatty acids, alpha-linolenic acid, genetic risk score, type 2 diabetes, interaction

## Abstract

Modulation of n-3 fatty acids on genetic susceptibility to type 2 diabetes (T2D) is still not clear. In a case-control study of 622 Chinese T2D patients and 293 healthy controls, a genetic risk score (GRS) was created based on nine T2D genetic variants. Logistic regression was used to examine the interaction of the GRS with erythrocyte phospholipid n-3 fatty acids for T2D risk. Every 1-unit (corresponding to 1 risk allele) increase in GRS was associated with 12% (Odds ratio (OR): 1.12; 95% confidence intervals (CI): 1.04–1.20) higher risk of T2D. Compared with the lowest quartile, participants had lower T2D risk in the 2nd (OR: 0.55; 95% CI: 0.36–0.84), 3rd (OR: 0.58; 95% CI: 0.38–0.88) and 4th (OR: 0.67; 95% CI: 0.44–1.03) quartile of alpha-linolenic acid (ALA) levels. Significant interaction (*p*-interaction = 0.029) of GRS with ALA for T2D risk was observed. Higher ALA levels were associated with lower T2D risk only among participants within the lowest GRS tertile, with ORs 0.51 (95% CI: 0.26–1.03), 0.44 (95% CI: 0.22–0.89) and 0.49 (95% CI: 0.25–0.96) for the 2nd, 3rd and 4th ALA quartile, compared with the 1st. This study suggests that higher erythrocyte ALA levels are inversely associated with T2D risk only among participants with low T2D genetic risk, with high genetic risk abolishing the ALA-T2D association.

## 1. Introduction

Prevalence of type 2 diabetes (T2D) is increasing globally, especially among developing countries, gaining higher public health priority for its prevention [1]. Evidence from several randomized controlled trials suggests that physical activity and dietary intervention could prevent or delay progression of T2D [2,3,4]. In addition, T2D is subject to genetic predisposition, with more than 70 genetic loci being identified [5]. Emerging evidence indicates that interaction of genetic variants with diet or lifestyle may play an important role in the development of T2D [6,7]. Specifically, genome-wide interaction of genotype by erythrocyte n-3 fatty acids has significant variance contributions for T2D traits, such as insulin resistance, fasting insulin and glucose [8]. In addition, one previous study suggests that erythrocyte total n-3 fatty acids interact with genetic variant at *PEPD* gene (encoding peptidase D, which plays an important role in the recycling of proline and collagen metabolisms) to modulate T2D risk in a Chinese population, with higher n-3 fatty acids abolishing the adverse effect of the risk allele at *PEPD* on T2D [9].

Erythrocyte n-3 fatty acids are established nutritional biomarkers of dietary intake of n-3 fatty acids [10]. There are two types of n-3 fatty acids, marine-source n-3 fatty acids (eicosapentaenoic acid (EPA), docosahexaenoic acid (DHA), docosapentaenoic acid (DPA)) and plant-based n-3 fatty acid (alpha-linolenic acid (ALA)). Although prior study has demonstrated an interaction between total erythrocyte n-3 fatty acids and *PEPD* variant on T2D, the interaction patterns among different types of n-3 fatty acids still not clear. On the other hand, genetic risk score (GRS) has been widely used in epidemiologic studies to investigate the interaction of environmental factors with genetic variation for certain disease outcomes, such as T2D and obesity [11,12,13,14]. So far, little is known about whether genetic susceptibility to T2D, captured by GRS, could be modified by n-3 fatty acids among Chinese populations. Therefore, the aim of the present study was to investigate the interaction of erythrocyte phospholipid marine and plant-based n-3 fatty acid with GRS, created based on several established T2D variants among East Asians, for T2D risk.

## 2. Materials and Methods

### 2.1. Study Population and Design

The study protocol was approved by the Ethics Committee of the College of Biosystem Engineering and Food Science at Zhejiang University (No. ZJU-BEFS-2013011) and the Institutional Review Board of Huadong Hospital (No. 2012023). All study participants gave informed written consent.

This was a case-control study, involving 622 T2D patients and 293 healthy controls. All the T2D patients were recruited from outpatient clinics across 30 hospitals in 20 provinces in China. The T2D patients were included if they met the World Health Organization criteria for the diagnosis of T2D (World Health Organization, 1999). Exclusion criteria were: (1) abnormal vitamin and mineral absorption (malabsorption syndrome); (2) use of vitamin and mineral supplementation within the last 3 months, so as to avoid the short-term influence of these supplements on the blood biomarkers and make it more comparable for the blood fatty acids among cases and controls (for example, T2D patients may be more prone to take supplements); (3) severe renal, hepatic, heart or psychiatric diseases (except for the diabetic complications); (4) a history of cancer, thyroid disease, alcohol abuse, pregnancy or lactation. Healthy controls were recruited via a health check program in Hangzhou, with the exclusion criteria: free of hypertension, renal disease, hyperlipidemia, hematological disorders, diabetes, family history of cardiovascular disease or diabetes, alcohol abuse and drug use.

### 2.2. Genotyping and GRS Creation

Nine independent single-nucleotide polymorphisms (SNPs) from nine different genomic regions with minor allele frequency >0.20 were selected for genotyping, with all these SNPs associated with T2D in discovered and replicated in East Asian populations (Chinese, Japanese or Korean) based on the National Human Genome Research Institute [15]. These nine SNPs were rs1436953 (*C2CD4A-C2CD4B*), rs11257655 (*CDC123*), rs4712524 (*CDKAL1*), rs2383208 (*CDKN2B*), rs16955379 (*CMIP*), rs3786897 (*PEPD*), rs831571 (*PSMD6*), rs13266634 (*SLC30A8*) and rs1359790 (SPRY2). Blood DNA was isolated by using the QIAamp DNA Blood Mini kits (Qiagen, Valencia, CA, USA). Then the selected SNPs were genotypes with TaqMan SNP genotyping kits on the ABI PRISMA 790HT Sequence Detection System (Applied Biosystems, Foster City, CA, USA), with an average genotyping success rate of 98%. Unweighted GRS based on the nine genotyped SNPs was generated by summing the number of effect allele for each SNP among the participants. We imputed the missing genotypes by assigning the mean risk allele frequency for a given SNP for cases and control separately, so as to maximize the sample size. This was done only for individuals successfully genotyped for at least eight of the nine SNPs. A weighted GRS was also generated, with the weights of each SNP being equal to the log-odds ratio for that SNP from original genome-wide association studies of East Asian populations (Table 1) [15].

### 2.3. Measurement of Erythrocyte Phospholipid Fatty Acid and Other Covariates

After an overnight fast, venous blood was collected by trained nurses with 21-gauge needles in plain and Ethylenediaminetetraacetic acid (EDTA) vacuum tubes, with red blood cells separated by standardized procedure. Erythrocyte membrane phospholipid fatty acids were measure using standard methods [16]. Briefly, 1 mL of distilled water was added into 200 μL erythrocytes and centrifuged for 10 min at 3000 rpm; then 1 mL of distilled water was added into the pellet (containing phospholipid membranes) which was centrifuged again. After centrifuging, total lipids were extracted with chloroform/methanol (1:1); and subsequently phospholipid fraction was separated by thin lay chromatography. Finally, fatty acid methyl esters were prepared and analyzed using gas chromatography [16].

Anthropometric parameters (including body weight, height and waist circumference), blood pressures and other lifestyle information were collected by a research assistant trained in standardized procedures after blood collection.

### 2.4. Statistical Analyses

All the statistical analyses were performed using STATA version 14 (StataCorp, College Station, Texas, TX, USA). Hardy-Weinberg equilibrium of the genotyped SNPs was examined with a Chi-square test. The difference in GRS values between control and T2D groups was compared with two-sample *t*-test. The associations of GRS with T2D risk, and erythrocyte phospholipid fatty acids with T2D risk were examined with a logistic regression model, adjusting for age and sex. In the primary analysis, logistic regression was used to examine the interaction of the unweighted GRS with erythrocyte marine n-3 fatty acids (C20:5n3 + C22:5n3 + C22:6n3) and alpha-linolenic acid (C18:3n3) for T2D risk, adjusting for age and sex. Fatty acids were categorized into two groups according to the population median. GRS was categorized as tertiles for the interaction analysis. Stratified analyses by GRS or by erythrocyte fatty acids were conducted if significant interaction was observed. We did not apply Bonferroni correction, as the present study is based on a hypothesis-driven approach to test the interaction with GRS for marine- and plant-based n-3 fatty acids, and actually only two interaction tests have been done in the primary analyses.

We then performed several sensitivity analyses: (1) We further adjusted body-mass index (BMI) in addition to age and sex in the primary interaction analysis; (2) We examined the interaction of weighted GRS with erythrocyte fatty acids for T2D risk, in order to investigate the influence of different weights/effect sizes of the SNPs on the results; (3) We examined the interaction of original unweighted GRS (created from original, non-imputed genetic variants) with erythrocyte fatty acids for T2D risk to test the influence of genotype imputation on the results. A two-tailed *p*-value < 0.05 was considered as statistically significant in the present study.

## 3. Results

### 3.1. Population Characteristics

The minor allele frequency of the nine genotyped SNP ranged from 0.29 to 0.44, and all SNPs were consistent with Hardy-Weinberg equilibrium (Table 1). The mean of the unweighted and weighted GRS was 11.1 (standard deviation (SD) = 2.06) and 10.8 (SD = 2.19) respectively. The GRS value in the healthy control group was significantly lower than that in the T2D group (*p* < 0.001). The population characteristics and erythrocyte phospholipid n-3 fatty acids by the unweighted GRS tertiles were presented in Table 2.

### 3.2. Association of GRS with T2D, and Erythrocyte Phospholipid n-3 Fatty Acids with T2D

Every 1-unit (corresponding to 1 risk allele) increase in unweighted GRS was associated with 12% (OR = 1.12; 95% CI: 1.04–1.20; *p* = 0.003) higher risk of T2D. The result was similar for the weighted GRS (OR = 1.12; 95% CI: 1.05–1.20; *p* = 0.001).

Compared with lowest quartile, participants had lower T2D risk in the 2nd (OR: 0.55; 95% CI: 0.36–0.84), 3rd (OR: 0.58; 95% CI: 0.38–0.88) and 4th (OR: 0.67; 95% CI: 0.44–1.03) quartile of ALA levels. Similarly, the 2nd (OR: 0.42; 95% CI: 0.27–0.66), 3rd (OR: 0.47; 95% CI: 0.30–0.74) and 4th quartile (OR: 0.41; 95% CI: 0.27–0.64) of marine n-3 fatty acids had lower risk of T2D compared with the 1st quartile.

### 3.3. Interaction of Erythrocyte Phospholipid Fatty Acids with GRS on T2D

**Figure 1:**
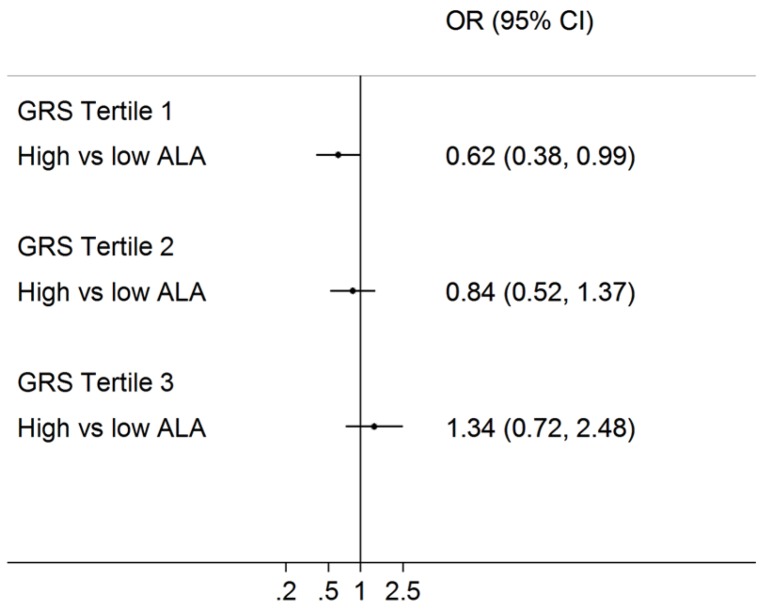
Association of erythrocyte alpha-linolenic acids (high vs. low levels) with risk of type 2 diabetes by the unweighted genetic risk scores. Logistic regression was used to estimate the interaction (*p*-interaction = 0.029) between unweighted genetic risk score (GRS) and erythrocyte ALA on T2D risk, adjusting for age and sex. ALA, alpha-linolenic acid. OR, odds ratio; CI, confidence interval.

There was significant interaction between erythrocyte ALA and unweighted GRS for the T2D risk (*p*-interaction = 0.029), adjusting for age and sex. The interaction remained significant when we further adjusted for BMI (*p*-interaction = 0.038) or when we used the original non-imputed GRS (*p*-interaction = 0.05). Briefly, high ALA levels, compared with low ALA levels, had significantly lower risk of T2D (OR = 0.62; 95% CI: 0.38–0.99) only among participants within the low GRS group (unweighted GRS tertile 1), but not for participants within the GRS tertile 2 or tertile 3 group (Figure 1). These significant associations still existed when ALA was categorized into quartiles. Higher ALA levels were associated with lower T2D risk only among the low unweighted GRS group (per quartile OR = 0.79; 95% CI: 0.64–0.97), but not among the other high GRS groups (Table 3). The results were similar, although non-significant (*p*-interaction = 0.21), for the weighted GRS. Higher ALA was associated with lower T2D risk among the GRS tertile 2 but not tertile 3 group.

On the other hand, when stratifying the participants into low and high ALA groups, we did not observe genetic associations (estimated from the unweighted GRS) in the low ALA group (per risk allele OR = 0.96; 95% CI: 0.74–1.25), while there was significant genetic association among the high ALA group (per risk allele OR = 1.43; 95% CI: 1.09–1.87) (Table 4). The results were similar for the weighted GRS.

## 4. Discussion

In the present study, we found that erythrocyte phospholipid ALA interacted with unweighted GRS to modulate the risk of T2D in a Chinese population. Briefly, the inverse association between erythrocyte phospholipid ALA and T2D only existed among individuals with low T2D genetic risk, but did not exist among those with high T2D genetic risk. We did not find interaction of GRS with marine n-3 fatty acids in the present study.

T2D is considered to result from the complex interplay between genetic and environmental factors [6]. Accumulating evidence supports the gene-environment interaction, especially after the advent of genome-wide association studies, which facilitated the investigation into the role of gene-environment interaction in the T2D etiology [6]. For example, TCF7L2 is one of the strongest T2D loci identified by genome-wide association studies, and several studies have shown that T2D risk associated with this gene could be modified by different dietary components, such as whole grain intake and carbohydrate quality and quantity [17,18]. With information from the genome-wide genetic variants, another study demonstrated the importance of genome-wide genotype-environment interaction in explaining the variance of diabetes-related traits [19]. GRS created based on the genetic variants identified by genome-wide associations studies has been used more and more widely as a proxy of genetic risk for certain traits/diseases, and to examine their interactions with diet in observational studies [11,12,13,14]. However, to the best of our knowledge, no previous studies have examined the interaction of T2D-related GRS with n-3 fatty acids for T2D outcome in Chinese populations.

Recent evidence suggests that blood marine n-3 fatty acids and plant n-3 fatty acid (ALA) showed heterogeneous associations with risk of T2D, with no significant association for marine n-3 fatty acids, but significantly inverse association for ALA [20]. The evidence was consistent with other trial evidence that ALA or flaxseed oil (rich in ALA) could improve glycemic traits [21,22,23,24]. Given the fact that the conversion of ALA to long-chain marine n-3 fatty acids was rather small (0.2%–8%) [25] and that no association with T2D was observed for marine n-3 fatty acids [20], some unique functions of ALA may exist linking to the etiology of T2D, independent of marine n-3 fatty acids. For example, ALA has been shown to induce insulin secretion through direct actions on G-protein receptors and stimulating enteroendocrine L-cells; and ALA could also enhance insulin sensitivity via regulating hepatic insulin-like growth factor-1 related pathways [26]. As the erythrocyte phospholipid ALA is an established biomarker of dietary intake of ALA [10,27], the present interaction observed for erythrocyte phospholipid ALA may reflect the influence of dietary ALA intake on the insulin sensitivity and secretion.

In the present study, consistent with the previous meta-analysis [20], we found an inverse association of erythrocyte ALA with T2D risk. Moreover, the association was modified by the genetic predisposition to T2D, with the significant association only existing among people with low T2D genetic risk (low GRS group). The mechanism behind the interaction was not clear. It may be that high GRS is related to the modification of some etiological pathways, thereby elevating T2D risk, which overlapped with the pathways linking ALA and T2D risk. Apparently, the current observation is quite novel and the speculation needs confirmation/replication in future studies. As another way to interpret the interaction, we stratified the association of GRS with T2D by the ALA median. We observed significant GRS-T2D association among participants with high ALA levels but not among those with low ALA levels. This result did not mean that low ALA could abolish the genetic effects. In contrast, participants with low ALA levels were already at high risk of T2D, while high GRS did not add additional risk for T2D.

We found significant interaction for unweighted GRS but not for weighted GRS. Weighted GRS takes different effect sizes of each SNP into consideration and is widely used together with the unweighted GRS [28]. We treated the results from unweighted GRS as the primary results as there was no large consortium-based meta-analysis effect size for the selected SNPs and the weights were derived from different genome-wide association studies with varied sample sizes. Therefore, unweighted GRS was a better option in this situation [28]. Nevertheless, we presented the results from weighted GRS as a sensitivity analysis, and the direction of the results was generally similar, although nonsignificant, for the unweighted GRS.

There are several limitations and strengths in the present study. First, we are the first to report the interaction between ALA and GRS on T2D risk, and the results lack replication in other independent studies. Second, sample size of the present study is moderate. Third, GRS is created based on only nine SNPs with high minor allele frequency (>0.2), which reduces the statistical power and is a major limitation of the present study. Much more T2D-related SNPs with lower minor allele frequency need to be considered in future studies with larger sample size. The main strength of the present study included the objective measurement of erythrocyte phospholipid fatty acids. Erythrocyte phospholipid n-3 fatty acids are the most suitable nutritional biomarkers for long-term dietary n-3 fatty acids, compared with plasma/serum lipid fractions [29,30].

## 5. Conclutions

In conclusion, the present study found a significant interaction between erythrocyte phospholipid ALA and T2D GRS to modulate the risk of T2D. The interaction suggests that, in order to prevent T2D, it may be more efficient to increase dietary intake of ALA in participants with lower T2D genetic risk. However, given the solid evidence of the beneficial associations of higher dietary/blood ALA with lower T2D and CVD, participants with low ALA status are recommended to increase dietary ALA intake regardless of their genetic profiles.

## Figures and Tables

**Table 1:** Characteristics of the nine selected single-nucleotide polymorphisms (SNP).

SNP Name	Chromosome	Region	Nearby Gene	*p*-HWE	MAF	Alleles (Minor Allele)	Risk Allele	Weight	Source
rs831571	3	3p14.1	*PSMD6*	0.057	0.347	C/T(T)	C	0.086	PMID: 22158537
rs4712524	6	6p22.3	*CDKAL1*	0.951	0.438	A/G(G)	G	0.199	PMID: 18711366
rs2383208	6	6p22.3	*CDKN2B*	0.903	0.401	A/G(G)	A	0.199	PMID: 18711366
rs13266634	8	8q24.11	*SLC30A8*	0.828	0.391	C/T(T)	C	0.199	PMID: 19401414
rs11257655	10	10p13	*CDC123*	0.249	0.419	C/T(C)	T	0.14	PMID: 22961080
rs1359790	13	13q31.1	*SPRY2*	0.563	0.290	C/T(T)	C	0.14	PMID: 20862305
rs1436953	15	15q22.2	*C2CD4A*, *C2CD4B*	0.587	0.343	A/G(A)	G	0.131	PMID: 21799836
rs16955379	16	16q23.2	*CMIP*	0.774	0.255	C/T(T)	C	0.077	PMID: 22158537
rs3786897	19	19q13.11	*PEPD*	0.729	0.440	A/G(G)	A	0.095	PMID: 22158537

HWE, Hardy-Weinberg equilibrium; MAF, minor allele frequency. Weight is the beta coefficient used to create genetic risk score, extracted from the source paper.

**Table 2:** Population characteristics by tertiles of the unweighted genetic risk score.

	Healthy Controls (*n =* 293)			Type 2 Diabetes Cases (*n =* 622)		
	GRS T1 (*n =* 119)	GRS T2 (*n =* 111)	GRS T3 (*n =* 63)	GRS T1 (*n =* 222)	GRS T2 (*n =* 228)	GRS T3 (*n =* 172)
	Median GRS *=* 9	Median GRS *=* 11	Median GRS *=* 13	Median GRS *=* 9	Median GRS *=* 11.9	Median GRS *=* 13.3
Age, years	51.8 (13.4)	50.5 (12.7)	50.8 (13.8)	58.7 (12.7)	59 (11)	59.2 (11.3)
Male, %	51 (43.6)	61 (55.5)	37 (58.7)	117 (52.7)	92 (40.4)	70 (40.9)
Height, cm	169.3 (8.3)	169.2 (8.1)	168.6 (8.7)	164.1 (8.5)	162.7 (7.6)	162.5 (8.7)
Weight, kg	69.4 (12.8)	70.9 (10.1)	71.3 (10.8)	68.5 (14.8)	66.2 (11.8)	65.3 (12.2)
BMI, kg/m^2^	24.1 (3.2)	24.6 (2.2)	25.0 (2.7)	25.3 (4.1)	24.9 (3.5)	24.7 (3.47)
DBP, mmHg	73.1 (13.9)	79.5 (12.6)	77.7 (10.2)	80 (10.1)	80.5 (11)	79.5 (11.7)
SBP, mmHg	122.9 (14.8)	128.1 (17.3)	128.5 (15)	131.2 (19.5)	130.4 (18)	128.9 (18.4)
Erythrocyte marine n-3 fatty acids (EPA + DHA + DPA), mol%	6.06 (4.03–7.76)	6.40 (3.93–8.22)	6.01 (4.01–8.68)	5.62 (3.46–6.4)	5.37 (3.2–6.5)	5.02 (3.1–6.03)
Erythrocyte EPA, mol%	1.75 (0.69–2.39)	1.77 (0.5–2.24)	1.90 (0.59–2.31)	1.76 (0.68–1.85)	1.76 (0.8–1.89)	1.58 (0.67–1.61)
Erythrocyte DHA, mol%	3.07 (1.52–5.06)	3.39 (1.54–5.2)	2.99 (1.72–3.84)	2.85 (1.65–3.62)	2.64 (1.31–3.39)	2.54 (1.55–3.21)
Erythrocyte DPA, mol%	1.25 (0.88–1.59)	1.24 (0.91–1.49)	1.13 (0.79–1.46)	1.01 (0.59–1.31)	0.97 (0.53–1.27)	0.90 (0.47–1.17)
Erythrocyte ALA, mol%	0.61 (0.25–0.70)	0.57 (0.25–0.50)	0.51 (0.19–0.53)	0.64 (0.19–0.57)	0.60 (0.19–0.57)	0.71 (0.21–0.67)

T, tertile; GRS, genetic risk score; BMI, body-mass index; DBP, diastolic blood pressure; SBP, systolic blood pressure; EPA, eicosapentaenoic acid; DHA, docosahexaenoic acid; DPA, docosapentaenoic acid; ALA, alpha-linolenic acid.

**Table 3:** Association of erythrocyte phospholipid alpha-linolenic acid with type 2 diabetes risk by the genetic risk scores.

GRS Categories	ALA Categories	Based on Unweighted GRS			Based on Weighted GRS		
		OR	95% CI	*p*	OR	95% CI	*p*
GRS Tertile 1	ALA Quartile 1	1	Reference		1	Reference	
	ALA Quartile 2	0.52	0.26–1.06	0.073	0.65	0.32–1.31	0.227
	ALA Quartile 3	0.42	0.21–0.85	0.016	0.52	0.26–1.04	0.066
	ALA Quartile 4	0.47	0.24–0.92	0.028	0.67	0.34–1.33	0.258
	Per Quartile	0.79	0.64–0.97	0.027	0.87	0.71–1.08	0.212
GRS Tertile 2	ALA Quartile 1	1	Reference		1	Reference	
	ALA Quartile 2	0.46	0.23–0.92	0.028	0.36	0.17–0.78	0.009
	ALA Quartile 3	0.61	0.3–1.23	0.165	0.52	0.24–1.16	0.112
	ALA Quartile 4	0.6	0.29–1.27	0.183	0.36	0.17–0.8	0.012
	Per Quartile	0.89	0.71–1.11	0.304	0.78	0.62–0.99	0.043
GRS Tertile 3	ALA Quartile 1	1	Reference		1	Reference	
	ALA Quartile 2	0.83	0.36–1.92	0.665	0.69	0.32–1.46	0.327
	ALA Quartile 3	0.9	0.38–2.14	0.812	0.66	0.31–1.42	0.291
	ALA Quartile 4	1.5	0.62–3.65	0.371	1.28	0.56–2.92	0.558
	Per Quartile	1.13	0.86–1.48	0.373	1.06	0.83–1.35	0.658

Logistic regression was used to estimate the association between erythrocyte ALA and risk of type 2 diabetes stratified by the genetic risk score (GRS) tertiles, adjusting for age and sex. ALA: alpha-linolenic acid.

**Table 4:** Association of genetic risk score with type 2 diabetes risk by the erythrocyte phospholipid alpha-linolenic acid levels.

ALA Categories	GRS Categories	Based on Unweighted GRS			Based on Weighted GRS		
		OR	95% CI	*p*	OR	95% CI	*p*
Low ALA group (≤median)	GRS Tertile 1	1	Ref.		1	Ref.	
	GRS Tertile 2	0.88	0.54–1.43	0.606	1.33	0.8–2.21	0.266
	GRS Tertile 3	0.93	0.54–1.6	0.794	1.35	0.81–2.24	0.251
	Per risk allele	0.96	0.74–1.25	0.763	1.16	0.9–1.5	0.249
High ALA group (>median)	GRS Tertile 1	1	Ref.		1	Ref.	
	GRS Tertile 2	1.29	0.81–2.06	0.289	1.4	0.85–2.3	0.185
	GRS Tertile 3	2.11	1.21–3.66	0.008	2.03	1.21–3.4	0.007
	Per risk allele	1.43	1.09–1.87	0.009	1.42	1.1–1.84	0.007

Logistic regression was used to estimate the association between genetic risk score (GRS) tertiles and risk of type 2 diabetes stratified by the erythrocyte ALA levels, adjusting for age and sex. ALA: alpha-linolenic acid.
